# Prosodic Cues to Word Order: What Level of Representation?

**DOI:** 10.3389/fpsyg.2012.00451

**Published:** 2012-10-30

**Authors:** Carline Bernard, Judit Gervain

**Affiliations:** ^1^Laboratoire Psychologie de la Perception (UMR 8158), Université Paris DescartesSorbonne Paris Cité, Paris, France; ^2^Laboratoire Psychologie de la Perception (UMR 8158)CNRS, Paris, France

**Keywords:** prosodic bootstrapping, word order, French, language acquisition

## Abstract

Within language, systematic correlations exist between syntactic structure and prosody. Prosodic prominence, for instance, falls on the complement and not the head of syntactic phrases, and its realization depends on the phrasal position of the prominent element. Thus, in Japanese, a functor-final language, prominence is phrase-initial, and realized as increased pitch (^ ***Tōkyō*** *ni* “Tokyo to”), whereas in French, English, or Italian, functor-initial languages, it manifests itself as phrase-final lengthening (*to* ***Rome***). Prosody is readily available in the linguistic signal even to the youngest infants. It has, therefore, been proposed that young learners might be able to exploit its correlations with syntax to bootstrap language structure. In this study, we tested this hypothesis, investigating how 8-month-old monolingual French infants processed an artificial grammar manipulating the relative position of prosodic prominence and word frequency. In Condition 1, we created a speech stream in which the two cues, prosody and frequency, were aligned, frequent words being prosodically non-prominent and infrequent ones being prominent, as is the case in natural language (functors are prosodically minimal compared to content words). In Condition 2, the two cues were misaligned, with frequent words carrying prosodic prominence, unlike in natural language. After familiarization with the aligned or the misaligned stream in a headturn preference procedure, we tested infants’ preference for test items having a frequent word initial or a frequent word final word order. We found that infants’ familiarized with the aligned stream showed the expected preference for the frequent word initial test items, mimicking the functor-initial word order of French. Infants in the misaligned condition showed no preference. These results suggest that infants are able to use word frequency and prosody as early cues to word order and they integrate them into a coherent representation.

## Introduction

The languages of the world show considerable variation in word order. In Japanese, for instance, the object precedes the verb [*ringo-wo taberu* (apple.acc^1^ eat) “eat an apple”] and postpositions follow their nouns [*Tokyo kara* (Tokyo from) “from Tokyo”] etc. In French, by contrast, the object follows the verb [*manger une pomme* (eat.inf^2^ an apple) “eat an apple”] and prepositions precede their nouns [*de Paris* (from Paris) “from Paris”]. As the examples suggest, this variation is not random: most languages conform to a basic word order type, which is usually characterized by the relative order of the object and the verb or by the typical position of function words within phrases (Greenberg, 1978; Dryer, 1992). Thus, Japanese is an OV or functor-final language, while French is VO or functor-initial. Crucially, the order of words in several phrase types correlates with that of the object and the verb. In OV languages, adpositions follow nouns, subordinate clauses precede the main verb and possessors precede the possessed. The opposite orders are observed in VO languages.

This knowledge is fundamental to language use, as it allows the efficient production and comprehension of multiword utterances. Indeed, infants know the basic word order of their mother tongue from their earliest multiword productions (Brown, 1973) and perceptually recognize word orders typical of their native language even earlier (e.g., Weissenborn et al., 1996; Höhle et al., 2001; Gervain et al., 2008). Importantly, the early mastery of word order might have a facilitatory effect on language acquisition, allowing young infants to correctly assign a grammatical function to novel structures or words they encounter.

How is word order learned? The purpose of the current paper is to contribute to a growing literature on the bootstrapping account of word order acquisition (Mazuka, 1996; Morgan and Demuth, 1996; Weissenborn et al., 1996; Gervain et al., 2008; Shukla and Nespor, 2010). Bootstrapping is a learning mechanism whereby the learner infers abstract, structural, perceptually unavailable properties of the target language on the basis of perceptually available cues in the input, which are correlated with the former (Morgan and Demuth, 1996). Under this view, the acquisition of a rudimentary, but already abstract representation of basic word order starts very early on, even before, and independently of the acquisition of a sizeable lexicon, on the basis of perceptually available cues such as word frequency and prosody, which correlate with word order. This bootstrapping account belongs to a larger family of theories on language development that assume language acquisition to rely on abstract structural representations from early on (Pinker, 1984; Gleitman et al., 1988; Fisher et al., 1991). These accounts contrast with the lexicalist view (Akhtar and Tomasello, 1997; Tomasello, 2000), according to which the knowledge of word order is initially linked to specific lexical items and becomes abstract only later, possibly only in the mature grammar.

Several recent studies have provided evidence that prelexical infants possess at least a simple representation of the basic word order of their native language. Specifically, two cues have been identified that infants might be able to exploit as indicators of the word order type of their mother tongue: word frequency and phrasal prosody.

Frequency-based word order bootstrapping relies on the observation that natural languages have two general word classes (Fukui, 1986; Abney, 1987): function words (articles: *the*, *a*, adpositions: *in*, *on*, *to*, pronouns: *he*, *she*, *they* etc.), indicating the morphosyntactic structure of sentences, and content words, carrying lexical meaning. Function words are typically more frequent than content words. Indeed, the 30–50 most frequent words are usually functors in all of the languages that have been studied in both adult- and child-directed speech (Kucera and Francis, 1967; Morgan et al., 1996; Gervain et al., 2008). Further, these frequent words often occupy utterance-initial and utterance-final positions, known to be perceptually salient and recognized even by young infants (Aslin et al., 1996). Importantly, the specific position they occupy correlates with word order: in OV languages, functors tend to appear phrase-finally, whereas they are phrase-initial in VO languages (Gervain et al., 2008). Thus tracking the most frequent words and their positions relative to salient utterance boundaries provides a cue to word order. It has been shown that 8-month-old monolingual Japanese and Italian infants are able to use this cue in an artificial grammar learning task to bootstrap the opposite word orders that characterizes their native languages (OV for Japanese, VO for Italian). In this study, infants were familiarized with an artificial grammar consisting of strictly alternating frequent and infrequent nonce words. As no phase-information is given (the beginning and the end of the stream are ramped in amplitude), the structure of this grammar is ambiguous between a frequent word initial (FI) and a frequent word final (FF) parse. In the test phase, infants are tested on their preference for FI and FF sequences. As predicted, Italian infants preferred the FI items, while Japanese babies looked longer at the FF items, reflecting the typical word order of these two languages. It is important to note that both FI and FF sequences were taken from the familiarization stream, so they were both familiar to infants. The only difference between the two groups that could explain the observed differences in their preferences during test was the opposite word orders of their mother tongues. This study thus shows that 8-month-old infants already have an expectation about the word order of their native language in terms of the relative position of frequent and infrequent words, and use it to parse a novel stream.

However, word frequency is not the only cue to word order (Morgan et al., 1996) and under some circumstances, it might not even be sufficient on its own. If an infant is exposed to a mixed language like German or Dutch, in which both OV and VO structures appear (German: (*weil ich) Papa sehe* because I Daddy see “because I see Daddy” and (*denn ich) sehe Papa* because I Daddy see “because I see Daddy,” Dutch: *op de trap* up the stairs “up the stairs” & *de trap op*), or to two languages with opposite orders, e.g., Japanese and Italian, then both FI and FF orders are found in the input she receives. Another well-established cue to word order, which can be used in combination with word frequency, is phrasal prosody (for a recent formulation of the proposal, see Shukla and Nespor, 2010). The prominence typically falls on the content word, i.e., the infrequent element, in prosodic phrases, hence its position correlates with word order. It is usually phrase-initial in OV or functor-final languages and phrase-final in VO or functor-initial languages (Nespor and Vogel, 1986). Even more importantly, the acoustic realization of phrasal prominence differs in these two positions, i.e., it correlates with word order. In OV languages, phrasal prominence is typically realized as increased pitch and/or intensity on the stressed vowel of the prominent word, so phrases tend to have a high-low or strong-weak pattern, whereas in VO languages, prominence is realized as increased duration on the stressed vowel of the prominent element, so phrases shown a short-long pattern (Nespor et al., 2008). Interestingly, this has been shown to hold true not only across languages, but also within a language, e.g., in the OV and VO phrases of German (Nespor et al., 2008). This differential acoustic realization means that there is a low-level, perceptually available cue in the input signal that correlates with word order. Further, it has been argued that these different acoustic features, i.e., pitch/intensity vs. duration, trigger different perceptual groupings. Known as the iambic-trochaic law (ITL, Hayes, 1995) and originally described for non-linguistic auditory stimuli (Bolton, 1894; Woodrow, 1951), this principle argues that elements contrasting in intensity or pitch are naturally perceived as having initial prominence, i.e., trochaic grouping, while elements contrasting in duration are perceived as prominence-final, i.e., iambic. This principle together with the different acoustic realization of prominence in OV vs. VO languages provides an automatic bootstrapping mechanism to cue word order (Mazuka, 1996; Nespor et al., 1996, 2008; Höhle et al., 2001; Shukla and Nespor, 2010).

Are infants able to exploit this cue? Sensitivity to prosody appears very early in development. Newborns’ communicative cries already show similarities with the prosodic patterns of the languages heard *in utero*, evidencing prenatal learning of prosody (Mampe et al., 2009). By 2 months of age, infants are able to discriminate the typical OV and VO prosodies described above (derived from Turkish and French, respectively), even when the stimuli are resynthesized to suppress all other distinctive features, e.g., segmental information (Christophe et al., 2003). Prosodic grouping preferences following the ITL have been documented as early as 6–8 months of age. Specifically, monolingual Japanese (OV) and monolingual English (VO) infants show language-specific prosodic grouping at 7–8 months, but not yet at 5–6 months (Yoshida et al., 2010) for the durational contrast with pure tone, i.e., non-linguistic, stimuli. Pitch and intensity were not tested in this study. For speech sequences, prosodic grouping was observed in monolingual Italian (VO) infants at 7 months with the pitch/intensity contrast, but not with duration (Bion et al., 2011). Differences in the nature and complexity of the stimuli used in the two studies might explain why a duration-based grouping preference was found in one VO-exposed population (English infants in the Yoshida et al., 2010 study), but not in the other (Italian infants in the Bion et al., 2011; study). Taken together, these studies suggest that prosodic grouping preferences start to emerge at around 7–8 months of age in the monolingual populations tested. Similar results were obtained when prosodic cues were combined with statistical information in a word segmentation task: 9-month-old infants were able to use intensity as a cue to word onset and duration as a cue to word offset with both pure tones and speech stimuli, while 6.5-month-old infants could only use the intensity cue, but not duration (Hay and Saffran, 2011).

Recently, infants’ ability to use prosody, and more specifically the ITL as a cue to word order has been tested directly (Gervain and Werker, under review). Seven-month-old OV (one of Japanese, Korean, Hindi/Punjabi, Farsi, or Turkish) – VO (English) bilinguals were exposed to a structurally ambiguous artificial grammar similar to the one used in Gervain et al. (2008). Importantly, prosody was added to the stream: half of the infants were exposed to the stream with OV prosody (pitch contrast), the other half to VO prosody (durational contrast). The test items were the same FI and IF sequences as in Gervain et al. (2008) with no prosodic cues (flat pitch and constant duration). Infants exposed to OV prosody showed a preference for the IF items, while infants in the VO prosody condition looked longer at the FI items. This suggests that OV–VO bilinguals are able to use phrasal prosody, in combination with word frequency, as a cue to select between the opposite word orders of their native languages. Interestingly, VO (English) monolinguals tested with the unfamiliar OV prosody did not show any preference, although they did prefer FI items when tested with no prosody, i.e., with only word frequency as a cue, replicating the monolingual Japanese and Italian findings (Gervain et al., 2008). This might indicate that by 7 months of age, monolinguals possess a stable representation of word order in terms of the distribution of frequent functors, which cannot be overridden by prosody when there is a conflict between the two cues (as was the case for the English monolinguals). An alternative explanation is that monolinguals may be less efficient at processing multiple cues, i.e., prosody and frequency, than bilinguals (Kovacs and Mehler, 2009a,b) and showed no preference in this task as a result of cognitive overload.

**Figure 1:**
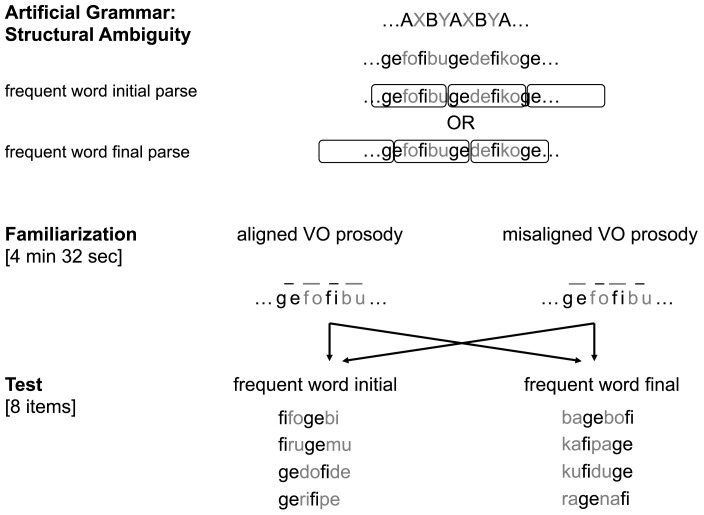
**The material used in Conditions 1 and 2**.

The current study, therefore, addresses two questions. First, we ask whether monolinguals are able to process word frequency and phrasal prosody simultaneously as cues to word order. Second, if they are, how do they integrate the two cues? To address these issues, we ran two studies (Figure 1), adapting the VO prosody condition from Gervain and Werker (under review). In Condition 1, the stimuli were identical to the VO prosody condition of Gervain and Werker (under review), with prosody and frequency perfectly aligned, i.e., with lengthening on the infrequent words as in natural language. We reasoned that for the monolingual French (VO) infants we tested, there is no conflict between prosody and frequency in this condition, so if they are able to process the two cues simultaneously, they should show a FI (VO) preference during test. If, however, the reason for their null preference in the Gervain and Werker (under review) study was the simultaneous presence of two cues, then they should also fail to show a preference in the present study. In Condition 2, we also used VO prosody and word frequency as cues, but now they were misaligned: prosodic prominence was shifted by one word, rendering the frequent words longer. This pattern, i.e., prosodic prominence on function words, is unusual in natural languages. Therefore, if infants integrate the two cues at the level of individual lexical items, then an ill-formed, misaligned representation arises, possibly disrupting infants’ preference for the FI (VO) pattern. If, however, prosody and frequency are processed separately, infants might still show a FI preference, because when considered independently, both cues are well-formed, native-like indicators of the functor-initial order of French.

## Materials and Methods

### Participants

Thirty (13 girls and 17 boys) 8-month-old (mean age: 8 months and 6 days, range: 6 months and 24 days to 8 months and 25 days) infants participated in Condition 1. Among these 30 children, five had one parent who spoke a language other than French: Arabic (2), Antillean Creole (1), Hungarian (1), Italian (1). Only the Italian-exposed infant was retained for analysis. Six other children did not complete the experiment because of fussiness and crying. Thus, 20 infants entered the analysis of Condition 1.

Another 36 (18 girls and 18 boys) 8-month-old (mean age: 8 months and 3 days, range: 6 months and 22 days to 8 months and 27 days) infants participated in Condition 2. Among these 36 children, seven had one parent who spoke a language other than French: English (1), Russian (1), Spanish (3), and Turkish (2). The Turkish and Russian-exposed infants were not retained for analysis. However, the English- and Spanish-exposed infants were, as both languages are VO with phrasal prosodies that are sufficiently similar to that French. In addition, 11 children did not complete the experiment because of technical problems (3), fussiness and crying (6), and too short or too long looking times (2). Since the duration of a test item was 960 ms and the maximum duration of a trial test was 21.84 s, we kept only the trials with fixation times strictly between these two values. Also, babies with more than two test trials rejected were not included in the final data analysis. Thus, 22 infants entered the analysis of Condition 2.

All parents gave informed consent before participation, and completed an information sheet.

### Material

An artificial grammar with ambiguous underlying structure was created for Conditions 1 and 2 (Figure 1), following Gervain and Werker (under review): a four-syllable-long basic unit AXBY was concatenated repeatedly. The A and B categories had one token each, while the X and the Y categories contained nine tokens, making individual X and Y tokens nine time less frequent than the A and B tokens. The lexicon of the artificial grammar consisted of the following words: A: *fi*, B: *ge*, X: *ru, pe, du, ba, fo, de, pa, ra, to*, Y: *mu, ri, ku, bo, bi, do, ka, na, ro*. This basic structure gave rise to a continuous stream of strictly alternating frequent (A and B) and infrequent (X and Y) words, mimicking function words and content words, respectively. The initial and final 15 s of the stream were ramped in amplitude in order to mask any phase-information. The familiarization stream was thus ambiguous between a frequent word initial or frequent-infrequent (e.g., AXBY) and a frequent word final or infrequent-frequent (IF; e.g., XBYA) parse.

The familiarization stream was synthesized using the fr4 female diphone database of MBROLA (Dutoit, 1997). In the two conditions, we used the same pitch (200 Hz) for all syllables (both frequent and infrequent words). We added native prosody (VO prosody) to the stream. We manipulated the relative position of prosodic prominence and word frequency. In Condition 1, the two types of cues were congruent: the non-prominent frequent words were short (240 ms) and the prominent infrequent words were long (320 ms). In Condition 2, we misaligned word frequency and word length so that frequent words were long (320 ms) and infrequent words were short (240 ms). The total duration of the two types of familiarization streams was 4 min 32 s.

The test items were eight four-syllabic chunks from the stream. Four of them instantiated the frequent-infrequent (FI) order (corresponding to a VO language; *fifogebi/firugemu/gedofipe/gerifipe*), the other four the IF order (corresponding to an OV language; *kafipage/kufiduge/bagebofi/ragenafi*). The prosody was flat for all the test items: with a constant 240 ms syllable duration, resulting in 960 ms long test items.

### Procedure

Participants were tested individually in a sound-attenuated room, with a low light intensity. The Headturn Preference Procedure (HPP, KemlerNelson et al., 1995) was used. Babies were seated on their caregiver’s lap in front of a central attention-getter light. Each experimental session consisted of a familiarization phase (with one of the two streams: word length and word frequency aligned or misaligned) immediately followed by a test phase. During the familiarization phase, a continuous stream, which lasted 4 min 32 s, was presented to the participants from two side speakers, associated with two attention-getter lights. During the familiarization phase, the lights were contingent upon the infants’ looking behavior, but were independent of the sound stimuli. During test trials, babies heard one of the eight four-syllabic chunks from the stream (four per condition). Before each test trial, infants’ attention was drawn to the central attention-getter light. Once this was achieved, the central light was turned off, and one of the sidelights was turned on. A test trial began when infants turned away from the central light and attended to the flashing sidelight. The test item was then presented at the same side. When babies looked for the maximum duration of the trial or if they looked away for more than 2 s, the trial ended, the sidelight was turned off, and the central attention-getter light started blinking again.

Each child heard eight test items: four in each condition (FI or IF). Stimuli were pseudo-randomized for each participant: there could not be more than two consecutive test items in the same condition. They were also counterbalanced between participants.

An experimenter observed infants’ behavior on a video monitor placed outside the experimental booth and controlled the lights and the stimuli. She listened to masking music and was blind to the stimuli being presented. Infants’ looking behavior was coded offline using the video recording made during the experiment.

## Results

**Figure 2:**
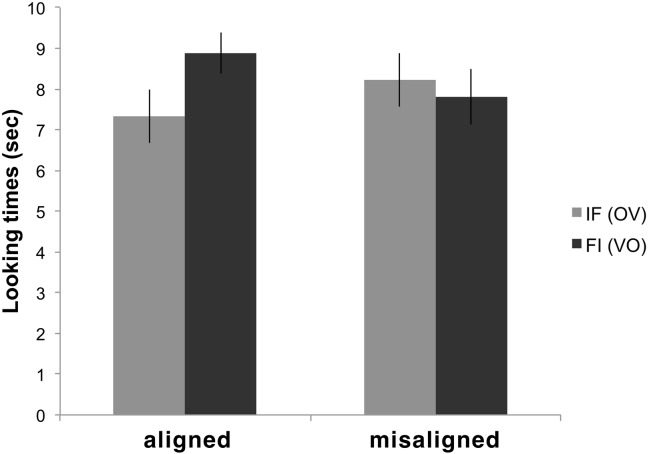
**The average looking times for FI and IF items in Conditions 1 and 2**.

The average looking times to FI and IF items in the two conditions are shown in Figure 2. We conducted an ANOVA with Familiarization Condition (Cond 1 aligned/Cond 2 misaligned) as a between-subjects factor and Test Item Type (FI/IF) as a within-subjects factor. We obtained a significant Familiarization Condition × Test Item Type interaction [*F*(1,40) = 5.3983, *p* = 0.026]. This was due to significantly longer looking times (Scheffe *post hoc* test *p* = 0.010) to *F*I test items than to IF ones in Exp 1 (aligned familiarization), but not in Exp 2 (misaligned familiarization). No other effect was significant.

## Discussion

In this study, we tested whether monolingual French-exposed 8-month-old infants are able to use word frequency and prosody as simultaneous cues to a rudimentary representation of the word order type of their native language. In an artificial grammar learning task, we found that they indeed showed the predicted preference for frequent word initial test items, mimicking the functor-initial word order of French, when the two cues were aligned at the level of lexical items, i.e., frequency words were non-prominent, but not when they were misaligned.

A possible alternative interpretation could be that infants in Condition 1 simply did not use prosody as a cue and succeeded on the basis of the frequency cue alone, as did monolingual Japanese and Italian infants in the Gervain et al. (2008) study. However, this interpretation is not probable, because if infants ignored prosody altogether in Condition 1, we would expect them to do the same in Condition 2, showing the same FI preference, contrary to fact.

Our results, therefore, suggest that monolinguals are not hindered by the presence of simultaneous cues as long as the prosodic cue is coherent with the frequency cue. This coherence is required at least at two levels. First, frequency and prosody cannot be in conflict: the OV prosody used with English-exposed infants in the Gervain and Werker (under review) study gives rise to a null preference, as neither cue overrides the other, i.e., they carry equal weight. Second, the prosodic cue and the word frequency cue need to be aligned at the lexical level, suggesting that the two cues are processed in an integrated manner.

What representations are formed through this integrative process? Further research is needed to explore the full details of how word order is acquired. It is not clear, for instance, whether both the frequent and the infrequent words are learned, or only the frequent ones. What the present study shows, however, is that infants expect lexical categories that follow the characteristics of those found in natural languages. Thus, they expect frequent words to occupy the typical positions of functors and to be prosodically less prominent than infrequent words, reflecting their knowledge of the typical features of functors, and content words. This is in accordance with previous results showing that infants as young as newborns are able to discriminate functors and content words on the basis of their different perceptual properties, and have expectations about their function and sentential position at an early age (Gerken et al., 1990; Gerken and McIntosh, 1993; Morgan et al., 1996; Shi et al., 1999, 2006; Shi and Werker, 2001, 2003; Höhle and Weissenborn, 2003; Hochmann et al., 2010). Further, this knowledge is abstract enough to allow generalization to a novel language, reflecting the existence of a representation of word order in terms of functor positions.

This simple representation of basic word order type in terms of function word position might be a first step in bootstrapping more complex word order phenomena and grammatical structure in general. During subsequent language development, infants might enrich this representation relying on several sources. They might be able to exploit the correlations that exist between the position of functors and other word order phenomena, such as the relative order of Verbs and their Objects, main and subordinate clauses etc (Kucera and Francis, 1967; Gervain et al., 2008). They might rely on their emerging vocabulary of object and action labels (Bergelson and Swingley, 2012) or their increasing understanding of intentionality (Csibra and Gergely, 2009) to determine the syntactic and semantic patterns of simple utterances in their input and generalize them to understand and produce more complex structures, as suggested by the semantic (Pinker, 1984) and syntactic bootstrapping hypotheses (Gleitman et al., 1988; Fisher et al., 1991).

If infants integrate word frequency and phrasal prosody at the level of lexical categories, as argued above, can we really conclude that this bootstrapping mechanism is prelexical and independent of vocabulary learning, as claimed before? In our view, this conclusion is justified for at least two reasons. First, infants’ knowledge appears to be category- and not item-based. There is nothing about the specific words used as frequent and infrequent items in our study that requires them to be prosodically weak or strong, respectively. It is infants’ knowledge about the lexical category of functors and content words in natural language that allows them to process the aligned grammar as well-formed and the misaligned one as ill-formed. Second, although recent results suggest that infants show evidence of word learning between 6–9 months of age (Bergelson and Swingley, 2012), at 8 months, the age tested in this study, they certainly do not yet have a sizeable lexicon. Therefore, they have no item-based knowledge in the sense of Tomasello (2000) that could support the word order representations we have uncovered in this study.

Taken together, our findings suggest that a first representation of a fundamental property of the native language, word order, is bootstrapped very early in development on the basis of perceptual cues such as word frequency and phrasal prosody. This early acquisition might have a cascading effect on the subsequent development of the native grammar and the lexicon.

## Conflict of Interest Statement

The authors declare that the research was conducted in the absence of any commercial or financial relationships that could be construed as a potential conflict of interest.

## Footnotes

^1^acc: accusative case

^2^inf: infinitive
